# Prevalence of soil-transmitted helminths and *Schistosoma mansoni* among a population-based sample of school-age children in Amhara region, Ethiopia

**DOI:** 10.1186/s13071-018-3008-0

**Published:** 2018-07-24

**Authors:** Andrew W. Nute, Tekola Endeshaw, Aisha E. P. Stewart, Eshetu Sata, Belay Bayissasse, Mulat Zerihun, Demelash Gessesse, Ambahun Chernet, Melsew Chanyalew, Zerihun Tedessse, Jonathan D. King, Paul M. Emerson, E. Kelly Callahan, Scott D. Nash

**Affiliations:** 1 The Carter Center, 453 Freedom Parkway, Atlanta, GA 30307 USA; 2 The Carter Center, P.O. Box 13373, Addis Ababa, Ethiopia; 3 Amhara Regional Health Bureau, P.O. Box 495, Bahir Dar, Ethiopia; 4 World Health Organization, Avenue Appia 20, 27 Geneva, Switzerland; 5 International Trachoma Initiative, 330 West Ponce de Leon Ave, Decatur, GA 30030 USA

**Keywords:** Soil-transmitted helminths, *Schistosoma mansoni*, School-age children, Prevalence, Infection intensity, Trachoma impact survey, Neglected tropical diseases

## Abstract

**Background:**

From 2011 to 2015, seven trachoma impact surveys in 150 districts across Amhara, Ethiopia, included in their design a nested study to estimate the zonal prevalence of intestinal parasite infections including soil-transmitted helminths (STH) and *Schistosoma mansoni*.

**Methods:**

A multi-stage cluster random sampling approach was used to achieve a population-based sample of children between the ages of 6 and 15 years. Stool samples of approximately 1 g were collected from assenting children, preserved in 10 ml of a sodium acetate-acetic acid-formalin solution, and transported to the Amhara Public Health Research Institute for processing with the ether concentration method and microscopic identification of parasites. Bivariate logistic and negative binomial regression were used to explore associations with parasite prevalence and intensity, respectively.

**Results:**

A total of 16,955 children were selected within 768 villages covering 150 districts representing all ten zones of the Amhara region. The final sample included 15,455 children of whom 52% were female and 75% reported regularly attending school. The regional prevalence among children of 6 to 15 years of age was 36.4% (95% confidence interval, CI: 34.9–38.0%) for any STH and 6.9% (95% CI: 5.9–8.1%) for *S. mansoni.* The zonal prevalence of any STH ranged from 12.1 to 58.3%, while *S. mansoni* ranged from 0.5 to 40.1%. Categories of risk defined by World Health Organization guidelines would indicate that 107 districts (71.3%) warranted preventive chemotherapy (PC) for STH and 57 districts (38.0%) warranted PC for schistosomiasis based solely on *S. mansoni*. No statistical differences in the prevalence of these parasites were observed among boys and girls, but age and school attendance were both associated with hookworm infection (prevalence odds ratio, POR: 1.02, *P* = 0.03 per 1 year, and POR: 0.81, *P* = 0.001, respectively) and age was associated with infection by any STH (POR: 1.02, *P* = 0.03). Age was also associated with reduced intensity of *Ascaris lumbricoides* infection (unadjusted rate ratio: 0.96, *P* = 0.02) and increased intensity of hookworm infection (unadjusted rate ratio: 1.07, *P* < 0.001).

**Conclusions:**

These surveys determined that between 2011 and 2015, STH and *Schistosoma mansoni* were present throughout the region, and accordingly, these results were used to guide PC distribution to school-age children in Amhara.

**Electronic supplementary material:**

The online version of this article (10.1186/s13071-018-3008-0) contains supplementary material, which is available to authorized users.

## Background

The World Health Organization (WHO) estimates approximately 1.5 billion people are infected with soil-transmitted helminths (STH) and that 207 million people currently have schistosomiasis, 90% of whom are living in sub-Saharan Africa [1, 2]. Recent studies, employing various methodologies to estimate national prevalences of STH and *Schistosoma mansoni* across Africa estimated Ethiopia to have the 13th highest prevalence of each disease group among over 40 countries [3, 4]. Within the Amhara region of Ethiopia, previously published surveys have reported community-level prevalence estimates as high as 65.6% for STH and 40% for *S. mansoni* [5–8]. While those surveys were small in scale, a larger, population-based survey in one central zone of Amhara found a prevalence of 19.5% for any STH and 2.3% for schistosomiasis among children aged from 2 to 15 years [9]. Prolonged infection with these parasites among children results in chronic malnutrition and anemia leading to stunted growth and impaired learning ability [10]. WHO guidelines for controlling morbidity caused by STH and schistosomiasis recommend distribution of preventive chemotherapy (PC) to school-age children in areas deemed to be at risk of these infections [11, 12].

Since 2007, the Amhara Regional Health Bureau’s Trachoma Control Programme has implemented the SAFE (surgery, antibiotics, facial cleanliness and environmental improvement) strategy to eliminate trachoma as a public health problem region-wide [13, 14]. The facial cleanliness and environmental improvement aspects of the SAFE strategy have the potential to impact many fecally transmitted parasitic diseases because of their focus on hygiene and sanitation. Previous research conducted in Amhara demonstrated that indicators of hygiene and sanitation had increased in the region since the initiation of the SAFE strategy and that SAFE interventions may have been a contributing factor to the apparent reduced prevalence of STH in parts of Amhara [9]. During that same timeframe, despite the existence of a national Enhanced Outreach Services Programme coordinating the distribution of albendazole, PC coverage in Amhara appeared to be inconsistent [9].

Recognizing the need to increase control efforts for STH and schistosomiasis in the region, the Amhara Regional Health Bureau leveraged trachoma impact surveys from 2011 to 2015, to produce population-based data on these parasitic diseases. The primary aim of these nested surveys was to provide regional and zonal estimates for STH and *S. mansoni* among school-age children in Amhara and to further describe their distributions by age, sex, and self-reported school attendance to inform distribution strategies. Upon completion of each survey round, prevalence data were reported to the Regional Health Bureau allowing them to make evidence-based programmatic decisions regarding PC distribution.

## Methods

### Sampling methodology and survey tools

Sampling methods for these surveys have been described elsewhere [9, 15]. Briefly, seven cross-sectional trachoma impact surveys took place semi-annually between 2011 and 2015. As part of these surveys, a subset of school-age children was randomly selected to allow for zonal estimates of STH and *S. mansoni*. The sample size for each zone was intended to determine a 25 ± 5% prevalence of intestinal helminths among school-age children at an alpha of 0.05. Furthermore, a design effect of 4.0 and a 10% non-response rate were assumed, resulting in a total of 1268 school-age children being targeted in each zone of Amhara. The number of individuals surveyed per district was determined based on the relative populations of each district.

Between 2011 and 2015, zones in eastern Amhara (Wag Hemra, North Wollo, Oromiya, South Wollo and North Shewa) were surveyed in the months of December, January and February, and zones in western Amhara (Awi, West Gojjam, East Gojjam, South Gondar and North Gondar) were surveyed in the months of May through August on account of the schedule for mass drug administration of antibiotics for trachoma in Amhara. Seven districts of North Gondar were surveyed in October 2015 during the last survey. During the survey, children were examined for trachoma and survey enumerators asked participating children about their daily habits including a self-report of school attendance.

For each zone, a multi-stage sampling procedure was used whereby villages (gotts) were selected with a probability proportional to estimated size. With the help of community leaders, villages were segmented into groups of approximately 40 households, a segment was randomly selected, and all households within each selected segment were surveyed. All households within each selected segment were eligible for inclusion in the survey evaluating prevalence of clinical signs of trachoma. Approximately 25 children (6 to 15 years) in each cluster were further selected to participate in a nested survey evaluating infection with intestinal parasites. When households included two or more children within the eligible age range, the survey software randomly selected one child to participate in this nested survey [16]. Assenting children were provided a clean stool container with a lid and wooden paddle and asked to provide a stool before the survey team left the household. From the provided stool, field technicians collected, weighed and preserved an approximately 1 g sample in 10–15 ml of sodium acetate-acetic acid-formalin (SAF) [17]. Preserved samples were then labeled and transported without refrigeration while protected from sunlight to the Amhara Public Health Research Institute in Bahir Dar, Ethiopia.

### Training, laboratory diagnostics and quality control

Before each survey round, data collection teams received in-class and field training on data and sample collection protocols. Preserved stool samples were processed using the ether concentration method and the resulting sediment was analyzed under a microscope at the Amhara Public Health Research Institute [17]. All sediment from the 1 g sample was examined regardless of the number of slides required to do so. Laboratory technicians received training on the ether concentration method, microscopically reading slides and identifying parasite eggs by species [9]. To ensure quality control during the identification process, every tenth negative specimen and every positive specimen was re-examined by a senior microscopist. Eggs of each species of helminth were counted and recorded unless the counts exceeded 100 eggs in which case, they were recorded as 100+ [18].

### Data management and statistical analysis

Survey data was collected electronically using custom built survey software [16]. Specimens were labeled with barcode stickers and scanned by the software linking each participant’s survey data to its corresponding stool samples. Laboratory results were recorded on paper by technicians and then two independent data entry clerks entered the data in Microsoft Access separately. Differences between the two data entry rounds were then reconciled using the appropriate hard copy. A stool sample being positive for any STH was defined as being positive for at least one egg of at least one of the following: *Ascaris lumbricoides*, *Trichuris trichiura*, and hookworm spp. (either *Anclystoma duodenale* or *Necator americanus*) [11]. Data was then analyzed in Stata 14 (StataCorp, College Station, TX, USA) using the svyset and svy commands to provide estimates and confidence intervals that account for the survey design by weighting observations by the inverse of their probability of selection. After the completion of each survey, results and data were reported to the Amhara Regional Health Bureau. For parasite prevalence, bivariate logistic regression was used to analyze associations with sex, self-reported school attendance (yes, no) and age in years. For infection intensity measured by egg count per gram of stool, the arithmetic mean and standard deviation (SD) are reported to describe the observed distributions and negative binomial regression was used to analyze associations with age, sex and self-reported school attendance [19].

## Results

**Fig. 1:**
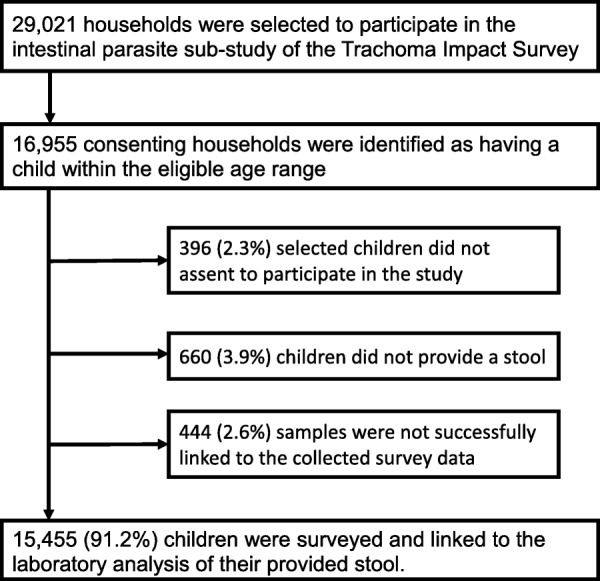
Flowchart displaying reasons for exclusion from the final sample

A total of 29,021 households were surveyed in 768 villages distributed across 150 districts in all ten zones of the Amhara region (Fig. 1). An eligible child was enumerated in 16,955 of the households. After the exclusion of children who did not assent, did not provide a sample, or whose sample could not accurately be linked to survey data, 15,455 (91.2%) children remained in the analyzed sample. The median age of children in the study was 10 years, 52.1% were female and 75.1% self-reported attending school (Table 1). The age and sex distributions of this study sample were similar to the distributions among all school-age children surveyed in the larger trachoma impact survey sample.

**Table 1:** Characteristics of participants from the primary and nested surveys, Amhara region, Ethiopia, 2011–2015

Characteristic	Trachoma impact surveys		Nested surveys	
	*n*	%	*n*	%
Age, years				
6	8551	10.3	1584	10.3
7	11,062	13.3	2185	14.1
8	9873	11.8	2018	13.1
9	10,834	13.0	1619	10.5
10	8695	10.4	1845	11.9
11	4960	5.9	1056	6.8
12	9464	11.4	1815	11.7
13	6993	8.4	1353	8.8
14	6069	7.3	1020	6.6
15	6798	8.2	960	6.2
Female	41,417	50.1	8041	52.1
Attends school	43,623	68.5	11,479	75.1

### Prevalence

**Fig. 2:**
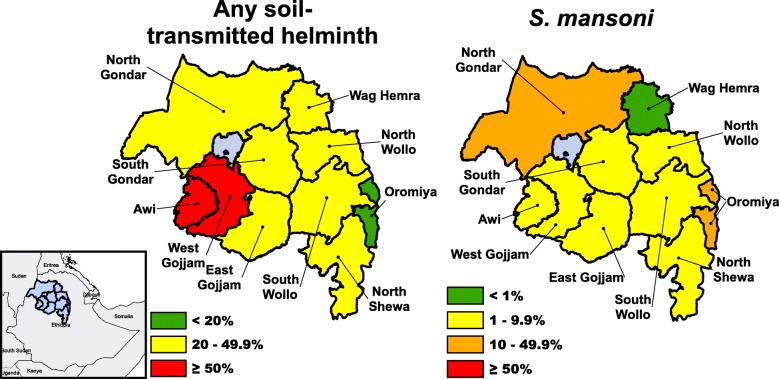
Zonal prevalence categorized by WHO guidelines for preventive chemotherapy in Amhara, Ethiopia, 2011–2015

Regional prevalence estimates for any STH and *S. mansoni* were 36.4% (95% confidence interval, CI: 34.9–38.0%) and 6.9% (95% CI: 5.9–8.1%), respectively. Zonal estimates for any STH ranged from 12.1% (95% CI: 7.5–19.0%) in Oromiya to 58.3% (95% CI: 52.2–64.1%) in Awi, while *S. mansoni* was lowest in Wag Hemra zone (0.5%, 95% CI: 0.1–2.1%) and highest in Oromiya zone (40.1%, 95% CI: 31.2–49.8%) (Table 2). Two of the ten zones had a prevalence of STH indicating they were high-risk areas for STH determined by the current WHO guidelines (≥ 50%), seven zones were low-risk areas (≥ 20% to < 50%), and one zone was below the 20% threshold of risk (Fig. 2). Although no zones had a prevalence of *S. mansoni* that was considered high-risk (≥ 50%), two zones were considered moderate-risk (≥ 10% to < 50%), seven zones were considered low-risk (≥ 1% to < 10%), and one zone had a prevalence ≥ 1%. Both STH and *S. mansoni* were equally prevalent among boys and girls (Additional file 1: Table S1, Table 3). Age was significantly associated with STH (prevalence odds ratio, POR: 1.02; 95% CI: 1.00–1.03, per year), but not *S. mansoni*. School attendance was not significantly associated with either STH or *S. mansoni.*

**Table 2:** Zonal prevalence by parasite in Amhara, Ethiopia, 2011–2015

Zone	Survey years	Districts	Clusters	*N*	*A. lumbricoides*	*T. trichiura*	Hookworm	Any STH	*S. mansoni*
					% (95% CI)	% (95% CI)	% (95% CI)	% (95% CI)	% (95% CI)
Awi	2013	11	45	941	21.3 (15.8–28.0)	10.8 (7.5–15.3)	43.4 (37.1–49.8)	58.3 (52.2–64.1)	5.5 (3.5–8.6)
East Gojjam	2013	18	90	2048	10.3 (7.3–14.2)	3.8 (2.3–6.3)	26.6 (22.7–31.1)	37.6 (33.7–41.6)	1.0 (0.3–3.3)
North Gondar	2012–15	23	117	2497	26.2 (21.6–31.4)	0.6 (0.3–1.1)	24.1 (20.5–28.0)	45.8 (40.8–50.9)	22.8 (18.3–28.1)
North Shewa	2013–15	24	99	1920	16.0 (13.0–19.5)	2.2 (1.3–3.5)	10.4 (7.3–14.7)	25.9 (22.7–29.3)	1.2 (0.6–2.3)
North Wollo	2013–14	12	72	1522	22.5 (18.4–27.2)	8.2 (5.9–11.4)	4.4 (3.2–6.1)	30.9 (26.2–36.0)	2.5 (1.2–5.2)
Oromiya	2013–14	7	25	430	7.7 (4.0–14.6)	2.8 (1.5–5.2)	2.5 (1.1–5.4)	12.1 (7.5–19.0)	40.1 (31.2–49.8)
South Gondar	2011	10	99	1090	10.3 (7.5–13.9)	3.1 (1.9–5.0)	10.4 (7.2–14.7)	20.3 (16.3–25.0)	3.9 (1.5–9.7)
South Wollo	2013–15	22	119	2820	15.3 (13.2–17.7)	2.9 (2.0–4.2)	9.1 (7.7–10.8)	25.9 (23.5–28.3)	2.6 (1.6–4.1)
Wag Hemra	2013–14	7	28	578	19.7 (12.9–29.0)	4.6 (2.5–8.5)	7.6 (3.5–15.6)	30.0 (22.1–39.3)	0.5 (0.1–2.1)
West Gojjam	2012–13	16	74	1609	11.5 (8.7–15.1)	3.3 (2.2–4.9)	48.2 (43.1–53.4)	56.0 (51.4–60.6)	1.3 (0.4–4.0)

**Table 3:** Unadjusted prevalence odds ratios (POR) accounting for survey design

	*A. lumbricoides*		*T. trichiura*		Hookworm		Any STH		*S. mansoni*	
	POR	95% CI	POR	95% CI	POR	95% CI	POR	95% CI	POR	95% CI
Age (years)	1.00	0.98–1.02	1.03	1.00–1.07	1.02	1.00–1.04	1.02	1.00–1.03	1.02	0.99–1.05
Female	1.04	0.94–1.16	1.04	0.85–1.28	1.04	0.94–1.15	1.04	0.96–1.13	0.97	0.81–1.15
School attendance	1.04	0.88–1.22	1.08	0.81–1.44	0.81	0.71–0.92	0.92	0.82–1.03	0.77	0.57–1.04

Regional prevalence estimates for *A. lumbricoides*, *T. trichiura* and either hookworm species were 16.8% (95% CI: 15.4–18.3%), 3.8% (95% CI: 3.3–4.5%) and 20.6% (95% CI: 19.2–21.9%), respectively. Zonal estimates for each parasite ranged as follows: *A. lumbricoides* (7.7–26.2%), *T. trichiura* (0.6–10.8%) and hookworms (2.5–48.2%). Distributions of individual parasite prevalence were similar by sex but school attending children had significantly less hookworm than their non-attending peers (POR: 0.81; 95% CI: 0.71–0.92). Hookworm prevalence showed a significant association with age (POR: 1.02; 95% CI: 1.00–1.04, per year) but a similar association between *T. trichiura* and age was not significant (POR: 1.03; 95% CI: 1.00–1.07).

**Fig. 3:**
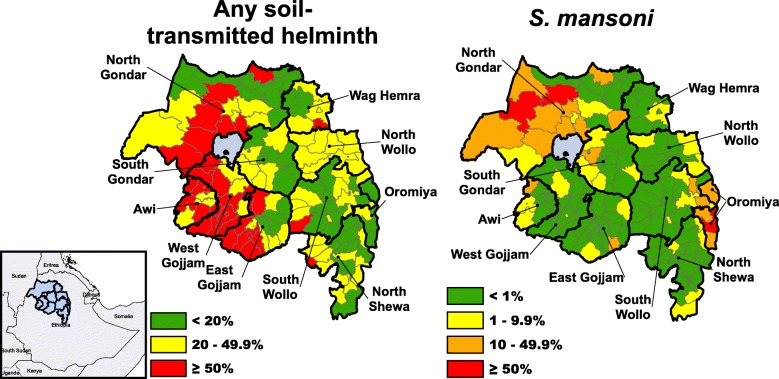
District risk level classified by estimated prevalence according to WHO guidelines for soil-transmitted helminths (left), and *S. mansoni* (right) in Amhara, Ethiopia, 2011–2015

None of the 150 districts were free from all three of the STH parasites. Following the decision by the Amhara Regional Health Bureau to base PC decisions on district prevalence, 37 districts (24.7%) required semi-annual treatment of school-age children with PC for STH and 70 districts (46.7%) required annual treatment of school-age children (Fig. 3). Based solely on the prevalence of *S. mansoni*, annual PC distribution to all school-age children was required in 7 districts (4.7%) while 20 districts (13.3%) required biennial PC distribution to this population. Thirty districts (20.0%) only required PC distribution for schistosomiasis twice during a child’s primary school years.

### Intensity

**Fig. 4:**
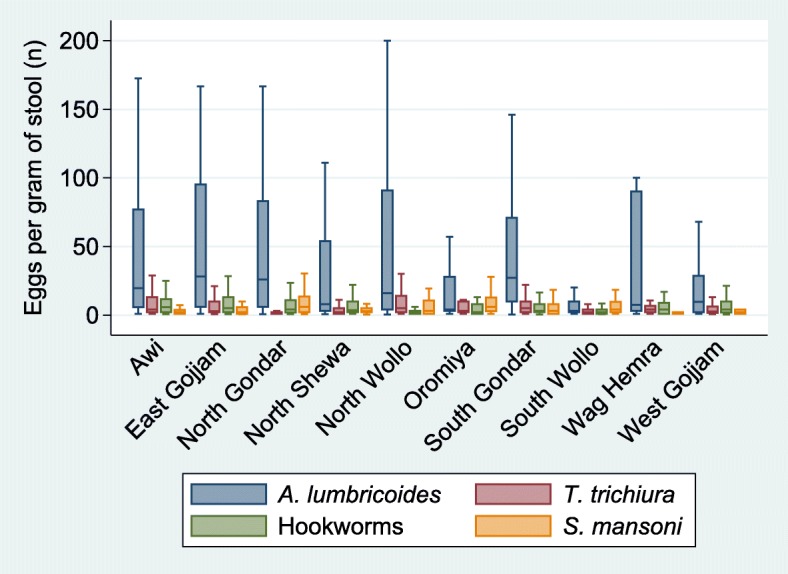
Distribution of infection intensities (eggs per gram of stool) by zone in Amhara, Ethiopia, 2011–2015

Among positive individuals, the regional mean egg count per gram of stool was 46.2 (95% CI: 37.2–55.2; SD: 4.6) for *A. lumbricoides*, 10.5 (95% CI: 6.9–14.0; SD: 1.8) for *T. trichiura*, 10.5 (95% CI: 9.5–11.5; SD: 0.5) for hookworms and 12.3 (95% CI: 9.4–15.1; SD: 1.5) for *S. mansoni*. Distributions of infection intensity among positive individuals by zone indicate that *A. lumbricoides* infections are more intense in most zones than infections by any other parasite (Fig. 4). All STH infections that did not reach the 100 egg count limit were low intensity infections (*A. lumbricoides*: < 5000, *T. trichiura*: < 1000, hookworms: < 2000 eggs per gram, epg). Samples that reached the 100 egg count limit represented 19.8%, 3.1%, 1.9% and 2.3% of the infections for *A. lumbricoides*, *T. trichiura*, hookworms and *S. mansoni*, respectively. After accounting for the mass of the sample, only 17 (1.8%) *S. mansoni* infections could be classified as at least moderate intensity (100–399 epg) [11]. Age was significantly associated with infection intensity (Table 4) for *A. lumbricoides* (unadjusted rate ratio, RR = 0.96; 95% CI: 0.93–0.99, per year) and hookworm (RR = 1.07, 95% CI: 1.03–1.10, per year). Sex and school attendance were not statistically significantly associated with any parasite’s infection intensity.

**Table 4:** Unadjusted rate ratio (RR) of expected eggs per gram accounting for survey design

	*A. lumbricoides*		*T. trichiura*		Hookworm		*S. mansoni*	
	RR	95% CI	RR	95% CI	RR	95% CI	RR	95% CI
Age, year	0.96	0.93–0.99	1.03	0.96–1.12	1.07	1.03–1.10	0.99	0.95–1.03
Female	0.98	0.72–1.34	1.35	0.82–2.21	0.96	0.80–1.14	0.93	0.64–1.34
School attendance	1.24	0.87–1.78	1.36	0.84–2.21	0.87	0.70–1.07	0.67	0.40–1.13

## Discussion

In a region-wide population-based sample of school-age children, the regional prevalence of STH (36.4%) and *S. mansoni* (6.9%) was low in Amhara as classified by WHO guidelines for helminth control. Furthermore, the estimated parasite prevalence placed the majority of the ten zones in the low risk categories, and only a small percentage of children had high intensity infections region-wide. The low prevalence of these parasites in Amhara may have resulted from sporadic deworming campaigns, sanitation and hygiene interventions conducted as part of the SAFE strategy, or to a secular trend. Despite the low parasite burden overall in Amhara, PC distribution was required in 107 districts (71.3%) for STH and 57 districts (38.0 %) for schistosomiasis based on *S. mansoni*. At the completion of each survey, these data were provided to the Amhara Regional Health Bureau for use by PC distributing programmes. Although WHO guidelines refer to “ecological zones” as the suggested unit of monitoring, future surveys in regions like Amhara should consider district level estimates to make PC distribution decisions easier for health officials.

Parasite prevalence estimates from Amhara were, in general, somewhat higher than the results of previous reports from Ethiopia. Two recently published systematic reviews have estimated the Ethiopian national prevalence of STH and *S. mansoni* to be 28.8 and 8.9%, respectively [3, 4]. In another recent study, Grimes et al. [20] surveyed schools in all regions of Ethiopia except Amhara and reported a prevalence of 13.3%, 7.8%, 7.4% and 3.5% for *A. lumbricoides*, *T. trichiura*, hookworms and *S. mansoni,* respectively. Differences between our results and these previous reports could be owing to geographical differences between Amhara and other regions. Rivers and lakes in Amhara are known to harbor snails suitable as intermediate hosts for multiple species of *Schistosoma* and southwestern zones are known for their higher concentration of agriculture possibly favoring hookworm transmission. These differences could also be owing to differences in season of data collection (rainy *vs* dry) and different diagnostics [21]. Our study utilized the SAF preservation and ether concentration method, while Grimes et al. [20] used the single slide Kato-Katz method which has shown a comparable sensitivity in low intensity settings for these STH except for *T. trichiura* (Kato-Katz: 69.0%, SAF: 21.5%) [22]. Conversely, the SAF method has been shown to have higher sensitivity (83.3%) for *S. mansoni* compared to the single slide Kato-Katz (70.7%) [23]. The SAF and ether concentration method were chosen because it allowed for the transport of a large number of samples from remote locations across the region to a laboratory where technicians could assay the samples under proper quality assurance.

From 2011 to 2015, the Trachoma Control Programme in Amhara successfully integrated the collection of stool samples into trachoma impact surveys to provide population-based parasite prevalence data to the Regional Health Bureau. These surveys were conducted after an average of approximately five years of the SAFE strategy which included the promotion of facial cleanliness through education campaigns and the promotion of latrine building. An earlier survey conducted in South Gondar zone of Amhara, demonstrated statistically significant increases in latrine usage, water availability and the frequency of face washing since the initiation of SAFE [9]. Along with documented sporadic albendazole distribution, these improvements may have played a role in a decreased prevalence of these parasites over time [9, 24]. Recently, however, a secondary analysis using a large subset of the survey data presented here, found no evidence of a protective association between community sanitation usage and STH infection [15]. Nevertheless, the results of these surveys demonstrated that a majority of the region fell under the low WHO risk category, despite the absence of a comprehensive deworming strategy. By adding stool sample collection to an existing trachoma monitoring programme, up-to-date district level data was made available to the Amhara Regional Health Bureau to begin planning PC distribution. To better inform coordination efforts between control programmes for neglected tropical diseases (NTD), future cost-benefit analyses of integrating community-based assessments for intestinal parasites into established community-based assessments for trachoma may be useful.

Males and females had a similar parasite prevalence in Amhara and only modest differences by age were observed. Although several previous studies conducted in Ethiopia reported conflicting evidence on the association between sex and prevalence of these parasites [25, 26], the size, geographical coverage and representative sampling strategy of this data would suggest that this association is not generalizable. We observed statistically significant associations between age and both combined STH prevalence and hookworm prevalence; however, both associations had a small effect size. In the infection intensity models, a 4% (95% CI: 1–7%) decrease in *A. lumbricoides* eggs per gram of stool was observed with every year increase in age, while conversely a 7% (95% CI: 3–10%) increase in hookworm eggs per gram of stool was observed. A child’s exposure to hookworm may increase with age as they travel further distances by foot and spend more time farming, while children may be exposed to *A. lumbricoides via* fecal-oral transmission at a younger age. A recent study in several communities in East Gojjam zone in Amhara found similar results, demonstrating that children 0–5 years of age had a higher prevalence of *A. lumbricoides* (10.8%) than hookworm infections (0.0%) [27]. Targeting one particular age group among school-age children in Amhara appears unwarranted given the modest differences in prevalence by age.

Heavy intestinal helminth infections are associated with reduced learning ability which could potentially impact school attendance [10, 28]. In Amhara, non-school attendees had a higher hookworm prevalence which could have been a result of deworming campaigns using school-based distribution strategies. Children that do not report attending school could also have a higher exposure to hookworm because they may spend more time performing agricultural work. Because of the cross-sectional nature of these surveys, determining the causal relationships between hookworm infection and self-reported school attendance was not possible. A recent Cochrane Review of deworming programmes in schools found limited and inconsistent findings for an effect of these programmes on school attendance [29]. However, since approximately 25% of surveyed children self-reported to not attend school, school-based interventions may miss an important subgroup of individuals, compromising the effectiveness of those programmes.

The results of this survey should be considered in light of several limitations. Mass drug administration of azithromycin for trachoma occurs at two different times of year for the eastern and western zones of Amhara. As a result, these survey data were collected in a manner that directly correlated geography within the region with seasonality of data collection. This aspect of the survey design makes comparisons between seasons impossible within any single zone. Future STH and *S. mansoni* research in the area should seek to sample at different times of year to better understand seasonal prevalence trends for these parasites in Amhara. The SAF preservation and ether concentration methods utilized are not recommended by the WHO; however, every method including Kato-Katz has shown a reduction in sensitivity for detecting infections in low-intensity samples [22]. Although SAF has shown a competitive sensitivity with other methods, the use of a single sample could have reduced parasite detection. Also, because of the 100 egg count limit and preferred size of a one gram stool sample, only light intensity infections for the STH species were likely to be classified. Despite this limitation, the low proportion of positive stools (10.0%) that reached the 100 egg count limit shows that the large majority of STH and *S. mansoni* infections among school-age children in Amhara were low-intensity.

## Conclusions

In summary, there is likely a continued need for PC for STH and *S. mansoni* across the Amhara region, even though most of Amhara is not high-risk, as determined through our large population-based survey. Despite large observed differences in hookworm prevalence between children that do and do not report school attendance, only minor differences by age and school attendance were detected for STH collectively and *S. mansoni*, suggesting that targeting all school-aged children for PC distribution is warranted in this population. The utilization of an existing trachoma survey platform allowed for the collection of actionable data for the NTD control programmes in the Amhara region.

## Additional file

Additional file 1: **Table S1.** Prevalence estimates accounting for survey design stratified by age, sex and school attendance, Amhara, Ethiopia, 2011–2015. (DOCX 20 kb)

## Abbreviations

NTD: Neglected tropical diseases
PC: Preventive chemotherapy
POR: Unadjusted prevalence odds ratio
RR: Unadjusted rate ratio
SAFE: Surgery, antibiotics, facial cleanliness and environmental improvement
STH: Soil-transmitted helminths
WHO: World Health Organization
