# Supplementary material for: Prevalence of soil-transmitted helminths and Schistosoma mansoni among a population-based sample of school-age children in Amhara region, Ethiopia
Source: Parasit Vectors. 2018 Jul 24;11:431. doi: 10.1186/s13071-018-3008-0 (PMC6056938; doi:10.1186/s13071-018-3008-0)
Supplement: Supplementary file 1 — Table S1. Prevalence estimates accounting for survey design stratified by age, sex and school attendance, Amhara, Ethiopia, 2011–2015. (DOCX 20 kb) [file 13071_2018_3008_MOESM1_ESM.docx]

**Table S1. Prevalence estimates accounting for survey design stratified by age, sex and school attendance, Amhara, Ethiopia, 2011-1015.**

|  | ***A. lumbricoides*** | | ***T. trichiura*** | | | **Hookworm** | | **Any STH** | | | ***S. mansoni*** | | |
| --- | --- | --- | --- | --- | --- | --- | --- | --- | --- | --- | --- | --- | --- |
|  | **%** | **95% CI** | | **%** | **95% CI** | **%** | **95% CI** | | **%** | **95% CI** | | **%** | **95% CI** |
| **Age, year** |  |  | |  |  |  |  | |  |  | |  |  |
| 6 | 15.5 | (13.0-18.3) | | 3.8 | (2.5-5.8) | 18.1 | (15.7-20.7) | | 32.1 | (29.1-35.2) | | 7.5 | (5.5-10.0) |
| 7 | 15.8 | (13.7-18.2) | | 3.0 | (2.2-3.9) | 19.5 | (17.3-22.0) | | 35.2 | (32.6-37.9) | | 6.6 | (5.0-8.7) |
| 8 | 17.8 | (15.4-20.6) | | 3.4 | (2.5-4.7) | 21.2 | (18.9-23.8) | | 37.5 | (34.4-40.6) | | 6.1 | (4.8-7.8) |
| 9 | 18.6 | (16.3-21.1) | | 3.7 | (2.6-5.2) | 22.2 | (19.6-25.1) | | 39.0 | (35.9-42.2) | | 6.1 | (4.7-8.0) |
| 10 | 16.2 | (13.7-19.0) | | 3.6 | (2.7-4.8) | 19.9 | (17.5-22.5) | | 35.5 | (32.6-38.6) | | 6.3 | (4.9-8.1) |
| 11 | 19.5 | (16.6-22.8) | | 5.8 | (4.3-7.7) | 20.6 | (17.6-24.0) | | 38.7 | (34.9-42.6) | | 7.8 | (5.9-10.2) |
| 12 | 15.1 | (13.0-17.4) | | 3.6 | (2.7-4.8) | 19.6 | (17.2-22.2) | | 34.0 | (31.3-36.9) | | 7.4 | (5.7-9.6) |
| 13 | 18.9 | (16.0-22.1) | | 4.9 | (3.7-6.5) | 20.7 | (17.7-24.2) | | 39.1 | (35.6-42.8) | | 6.8 | (5.0-9.2) |
| 14 | 14.6 | (12.2-17.3) | | 3.6 | (2.5-5.2) | 23.4 | (20.1-27.1) | | 37.8 | (34.1-41.6) | | 7.9 | (6.0-10.4) |
| 15 | 16.5 | (13.4-20.0) | | 4.2 | (2.9-5.9) | 22.3 | (18.9-26.2) | | 38.2 | (34.1-42.4) | | 8.0 | (5.7-11.0) |
| **Sex** |  |  | |  |  |  |  | |  |  | |  |  |
| Male | 16.5 | (15.0-18.1) | | 3.7 | (3.1-4.5) | 20.2 | (18.8-21.8) | | 36.0 | (34.2-37.7) | | 7.0 | (5.9-8.3) |
| Female | 17.0 | (15.5-18.7) | | 3.9 | (3.2-4.7) | 20.9 | (19.3-22.6) | | 37.0 | (35.1-38.9) | | 6.8 | (5.7-8.2) |
| **School attendance** |  |  | |  |  |  |  | |  |  | |  |  |
| No | 16.4 | (14.2-18.9) | | 3.6 | (2.7-4.9) | 23.4 | (21.1-25.8) | | 38.1 | (35.6-40.7) | | 8.2 | (6.2-10.9) |
| Yes | 17.0 | (15.5-18.5) | | 3.9 | (3.3-4.5) | 19.8 | (18.4-21.2) | | 36.1 | (34.5-37.8) | | 6.5 | (5.5-7.6) |
